# Exosomes Derived From Macrophages Enhance Aerobic Glycolysis and Chemoresistance in Lung Cancer by Stabilizing c-Myc via the Inhibition of NEDD4L

**DOI:** 10.3389/fcell.2020.620603

**Published:** 2021-03-04

**Authors:** Huan Wang, Lie Wang, Haiyan Pan, Yaona Wang, Miao Shi, Hang Yu, Chaoye Wang, Xinfu Pan, Zhijun Chen

**Affiliations:** ^1^Department of Cardiothoracic Surgery, Zhoushan Hospital, Zhejiang University, Zhoushan, China; ^2^Department of Internal Medicine, Zhoushan Hospital, Zhejiang University, Zhoushan, China

**Keywords:** glycolysis, M2 macrophage-derived exosomes, NEDD4L, lung cancer, chemoresistance

## Abstract

As one of the most common and lethal cancer, lung cancer severely threatens the health of human. It has been reported that tumor-associated macrophages promote initiation, progression, as well as chemoresistance in human cancers. However, the underneath molecular mechanism that drives chemoresistance in lung cancer is yet not fully characterized. In this article, we demonstrated that M2 macrophage-derived exosomes (MDE) is the key factor to promote cisplatin-resistance in lung cancer. MDE exhibited high expression level of several miRNA including miR-3679-5p. Mechanistically, miR-3679-5p was delivered to lung cancer cells by MDE, downregulating the expression of a known E3 ligase, NEDD4L, which has been identified as a key regulator controlling the stability of c-Myc. Such decreased NEDD4L expression level resulted in the stabilization of c-Myc and elevated glycolysis. The enhanced glycolysis drives the chemoresistance in lung cancer. Taken together, our findings not only show that M2 macrophage induce chemoresistance in lung cancer through MDE mediated miR-3679-5R/NEDD4L/c-Myc signaling cascade, but also shed the light on the mechanism of the cross-talk between M2 macrophage and lung cancers. By pinpointing a potential novel survival signaling pathway, our data could provide a new potential therapeutic target for lung cancer treatment and management.

## Introduction

Despite the rapid progression in the research of novel therapies, lung cancer remains the most malignant as well as lethal cancer due to relapse and resistance to treatment (Global Burden of Disease Cancer Collaboration et al., 2015; Yuan et al., 2019). Chemoresistance resulted from aberrant glucose metabolism accounts for most drug resistance cases (Lin et al., 2019). One widely accepted hypothesis is that the reprogramming of glucose metabolism causes a wide range of physiological changes, including abnormal DNA repairing, enhanced autophagy, and increased exosomes secretion, in cancer cells (Hay, 2016). Targeting aberrant glucose metabolism has become a promising strategy to cure cancer.

It has been well studied that tumor-associated macrophages (TAMs) play vital roles in tumorigenesis by forming a supportive niche to favor tumor metastasis in microenvironment (TME) (Pollard, 2004, 2009; Joyce and Pollard, 2009; Ojalvo et al., 2009). In general, after maturation, macrophages could polarize into two distinct macrophages: M1 and M2 macrophages, among which M1-polarized macrophages could produce cytokines including IL12, TNFα etc. that have been suggested being pro-inflammatory and immune stimulatory. Meanwhile, they could be activated by different cytokines such as IFNγ as well (Qian and Pollard, 2010). On the other hand, M2-polarized macrophages could be activated by several Th2 cytokines including IL4, IL10, and IL13, which are actually thought to resemble the characters of TAMs (Sica et al., 2014, 2015; Caronni et al., 2015). TAMs have been shown involved in multiple aspects of tumorigenesis including proliferation, invasion, and metastasis. In addition, TAMs have been demonstrated to induce tumor-associated angiogenesis as well as the inhibition of antitumor immune response, which eventually leads to the progression of tumor (Hagemann et al., 2004; Pukrop et al., 2006; Vasiljeva et al., 2006; Wyckoff et al., 2007). Collectively, all the data implied that TAMs could function as an attractive therapeutic target for cancer therapy. One possible mechanism of how TAMs promote cancer metastasis as well as drug resistance is that the survival signals from TAMs were transmitted to cancer cells through macrophage-derived exosomes (MDE). In recent years, exosomes derived from macrophage have evoked increased interest in the field of cancer research. Exosomes are a type of extracellular vesicles with bilayer lipid membrane that contain protein, DNA, and RNA. The size of exosomes ranges from 50 to 100 nm. Exosomes are produced in the endosomal compartment and are constitutively secreted by fusion with cellular membrane (Pan and Johnstone, 1983; Raposo et al., 1996, 1997; Thery et al., 1999; Blanchard et al., 2002; van Niel et al., 2006). It has been widely reported that exosomes play key tumorigenesis, drug resistance, as well as metastasis by mediating material exchange between tumor and stromal cells (Taylor and Gercel-Taylor, 2011; Luga et al., 2012; Peinado et al., 2012), and between tumor and macrophages (Lan et al., 2019; Yu et al., 2019; Zhu et al., 2019; Kwon et al., 2020). However, the mechanism of MDE driving chemoresistance in lung cancer is still under investigated.

In this article, we demonstrated that M2a macrophage promotes lung cancer cell chemoresistance by delivering MDE. We also demonstrated that, by inhibiting the expression of NEDD4L in response to MDE, miR-3679-5p could indirectly stabilize the c-Myc protein, which in turn enhances aerobic glycolysis and eventually promotes chemoresistance in lung cancer.

## Materials and Methods

### Chemicals

All the chemicals utilized in this paper including Cisplatin (C2210000), GW4869 (D1692), 2-Deoxy glucose (2-DG, D8375), and MG132 (M8699) were purchased from Sigma-Aldrich (LAA21, St. Louis, MO, USA). The chemicals were reconditioned and applied in accordance to the manufacturers' protocols.

### Human Lung Cancer Specimen Collection

The study was performed by following the ethical guidelines approved by the Institutional Ethical Review Committee of Zhoushan Hospital, Zhejiang University (Zhoushan, China). Briefly, a total of 85 lung adenocarcinoma (LUAD) patients who received surgery between January 2010 and December 2011 at Zhoushan Hospital (Zhoushan, China) were enrolled in the study after written informed consents were given. The LUAD tissues and adjacent non-tumorous tissue counterparts were collected from patients during the surgical operation, then fixed and embedded in paraffin, and eventually be used for IHC analysis. Thirty pairs of tumor and adjacent normal tissues were removed surgically and snap frozen in liquid nitrogen. All tissue samples are preserved at −80°C until further analysis required.

### Immunohistochemistry (IHC)

Specimens were deparaffinized in xylene and then rehydrated in serial diluted ethanol. For antigen retrieval, the slides were boiled in 0.01M citrate buffer (pH 6.0) for 15 min in a microwave. In order to inactivate endogenous peroxidases, the slides were treated with 0.3% hydrogen peroxide for 10 min, and then blocked with 5% BSA in TBST for 1 h. The slides were then incubated with antibody against NEDD4L (13690-1-AP, Proteintech) or c-Myc (ab32072, Abcam) overnight on a rocker at 4°C. The slides were washed in TBST, then covered by secondary antibody at room temperature for 1 h. 3,3-diaminobenzidine (DAB) solution (Vector Laboratories, Burlingame, CA, USA) was dropped onto the sections to label the positively stained cells, after which the sections were counterstained slightly with hematoxylin. The intensity of immunoreactivity was graded based on an established method from a previous report (Schmidt et al., 2009): IRS (immunoreactive score) = staining intensity (SI) × percentage of positive cells (PP). The SI was categorized as 0, negative; 1, weak; 2, moderate; 3, strong. A protein with staining index ≥3 would be defined as overexpression. IRS ≥9 will be considered as a highly positive signal. The IRS score for the staining intensity ranges from 0 up to 12. The percentage of positive cells (PP) was defined as follow: 1: 0–10, 2: 11–50, 3: 51–80, 4: 81–100%. The staining score was assessed by two independent investigators. Sections of non-neoplastic brain tissues (He et al., 2012) and colon cancer tissues (Ren et al., 2020) were taken as positive control slide for NEDD4L and c-Myc staining, respectively. Matched negative controls were stained without primary antibody.

### Cell Lines and Cell Culture

Human THP-1 monocytic cells were cultured in RPMI 1,640 medium (Millipore Sigma) with 10% FBS (Gibco) supplement. Human A549 lung cancer and human embryonic kidney (HEK) 293 cells were grown in DMEM medium supplemented with 10% FBS. The cells were maintained at 37°C in an atmosphere containing 5% CO_2_. All the cell lines are available from the cell bank of Shanghai Biology Institute, Chinese Academy of Science (Shanghai, China).

### Polarization of Macrophages

Mn THP-1 cells were treated with 100 ng/ml phorbol-12-myristate-13-acetate (Millipore Sigma) for differentiation; the medium was changed every 2 days for a week. The resting differentiated macrophages were with IL-4 (30 ng/ml, Biolegend) for 24 h for M2 Macrophage polarization. The cells were then washed and cultured in fresh medium. The conditioned medium from the polarized macrophages was collected 24 h after incubation and immediately stored at −80°C.

### Isolation and Characterization of Exosomes

Cells cultured medium was centrifuged and cell debris was removed. The supernatant was filtered with a 0.22-μm PVDF filter. Exosomes were extracted from the cell supernatants with VEX Exosome Isolation Reagent (Vazyme, Nanjing, China) based on the manufacturer's instruction. Exosomes were reconstituted in PBS buffer and frozen at −80°C for later analysis. The morphology as well as size distribution of the extracted exosomes were determined by transmission electron microscopy (FEI Tecnai 12, Philips, The Netherlands) and flow nano analyzer. The size as well as concentration of exosome particle were determined with ZetaView Nanoparticle tracking system PMX 110 (Particle-Metrix, Meerbusch, Germany), the data was processed by software ZetaView 8.02.28. The protein expression levels of TSG101 and CD63 were analyzed using western blot.

### Exosomes Uptake Assay

Exosomes were labeled with PKH26 Red Fluorescent Cell Linker Kits (Sigma-Aldrich) to determine their internalization by AC16 according to the manufacture's instruction. Briefly exosomes were firstly diluted in Diluent C, then incubated with PKH26 dye at 25°C for 5 min. FBS was added afterward and the mixture sit for 1 min for excess dye binding. Then the product was washed in KSFM and centrifuged again at top speed at 4°C for 75 min. Positively labeled exosomes remains at the bottom after discarding the supernatant and were resuspended in KSFM. 1 μg of labeled exosomes were added to A549 cells cultured on round coverslips in 24-well plate and incubated in a cell incubator with 5% CO_2_ at 37°C overnight. After that, A549 cells were rinsed twice with PBS, then fixed by 4% paraformaldehyde for 10 min. Slides were mounted with DAPI-containing antifluorescence-quenching agent.

### MicroRNA Interference and Overexpression

2 × 10^5^ cells were seeded in six-well plates and cultured in a cell incubation with 5% CO_2_ at 37°C overnight. The cells were transfected with the one of the three reagents: miR-3679-5p mimic (50 nM, 5′-UGAGGAUAUGGCAGGGAAGGGGA-3′), inhibitor (50 nM, 5′- UCCCCUUCCCUGCCAUAUCCUCA-3′), or a negative control miRNA (50 nM, 5′-CAGUACUUUUGUGUAGUACAA-3′) by using Lipofectamine 2000 Reagent (ThermoFisher, Cat# 11668019).

### Lentivirus Preparation

The full-length coding sequence of human *NEDD4L or c-Myc* was cloned into pLVX-puro (Clontech, Palo Alto, CA, USA). The lentivirus was produced in 293T cells and packaging plasmids, psPAX2 as well as pMD2.G.

### Real-Time Quantitative PCR

Briefly, total RNA was extracted with TRIzol reagent (Thermo Fisher Scientific) following the manufacturer's manual. One microgram of total RNA was used to produce cDNAs using the Revert Aid^TM^ cDNA Synthesis Kit (Cat#K1622; Thermo) with a special stem-loop primer for isolating miRNA transcripts and a random primer for isolating gene transcripts, respectively. The expression levels of indicated miRNAs and genes were quantified by qRT-PCR with SYBR Green PCR Master Mix (Cat#K0223; Thermo). *U6* and *GAPDH* were used as internal controls for miRNA and gene expression normalization, respectively. The primers used in this study could be found in Supplementary Tables 1, 2. The qRT-PCR was performed on a 7,300 Real-Time PCR System (Applied Biosystems, Foster City, CA, USA). The 2 ^ΔΔCt^ relative quantification method was applied to determine relative expression levels of miRNAs and genes.

### Western Blotting

Whole cell lysate was prepared in ice-cold 1X RIPA lysis buffer (Millipore SIGMA). The total protein lysate concentration was determined using a BCA protein assay kit (Thermo Fisher Scientific) according to the protocols of manufacturers. Twenty-five micrograms of protein extracts were resolved on a 10 or 15% SDS polyacrylamide gel and electroblotted onto a nitrocellular membrane (Millipore). Membrane was blocked with 5% non-fat milk, then incubated with primary antibodies (Supplementary Table 3) at 4°C overnight. Horseradish peroxidase-conjugated secondary antibody (1:3000, Cell Signaling Technology) was used for chemiluminescent detection. Immunoreactivity signals were visualized using ECL chromogenic substrate (Bio-Rad Laboratories).

### Measurement of 2-NBDG Uptake

The A549 lung cancer cells were rinsed twice with PBS and cultured in glucose-free Krebs-Ringer buffer (KRB) at 37°C for 15 min, after which 100 μM 2-NBDG (Cat#N13195, Thermo Fisher Scientific) was added to the culture and cultured at 37°C for 45 min. The cells were rinsed with ice-cold KRB buffer to stop the reaction, then the fluorescent signal was determined by GloMax®-Multi+ Detection System (Promega, Madison, WI, USA) using 535 nm emission and 485 nm excitation filter set. The intensity was firstly normalized by the protein quantity, then normalized to the control group intensity, and the relative fluorescent ratiometric quantitation was exhibited as % of control group.

### Lactate Production Assay

The A549 lung cancer cells were firstly treated as needed in the article, then the cell culture medium was collected at the end of treatments. Lactate concentration was determined with a Lactate Assay Kit (Millipore SIGMA) following the manufacturer's instruction.

### Measurement of Energy Metabolism in Cells

A549 lung cancer cells were seeded in the lower chamber, and M2 macrophages were seeded in transwell inserts. After 2 days, A549 cells in the lower chamber were plated in microplates at a density of 4 × 10^4^ cells/well and cultured overnight. The oxygen consumption rate (OCR) and extracellular acidification rate (ECAR) of A549 cells were monitored with XF96 extracellular flux analyzer (Seahorse Bioscience, USA) following the manufacturer's instruction. For ECAR, the glyco-stress test kit (Seahorse Bioscienc), 10 mM of glucose, 50 mM of 2-[N-(7-nitrobenz-2-oxa-1,3-diazol-4-yl) amino]-2-deoxyglucose, and 2 μM of oligomycin were used. For OCR, the mito-stress kit (Seahorse Bioscience), 1.5 μM of fluoro-carbonyl cyanide phenylhydrazone, 2 μM oligomycin, and pre-mixed 1 μM of antimycin A with 100 nM of rotenone were used.

### Co-immunoprecipitation (Co-IP) Assay

Fresh whole cells lysate in Tween-20 Co-IP buffer were immunoprecipitated with anti-NEDD4L (Abcam, Ab240753), anti-c-Myc (Abcam, Ab32072) or control IgG (Santa Cruz Biotech., Santa Cruz, CA, USA) antibodies and protein A/G-agarose beads over night at 4°C. Precipitates were rinsed three times with ice-cold Co-IP buffer and subjected to western blot to determine the pull-down efficacy.

### Luciferase Assay

Wild-type (WT) or mutant 3′-UTR of *NEDD4L* was integrated into pGL3 Vector (Promega, Madison, WI). In order to validate the efficacy of miR-3679-5p targeting *NEDD4L* 3′-UTR, A549 cells were transfected with negative control (NC) or miR-3679-5p mimic, WT or mutant pGL3-NEDD4L 3′-UTR plasmid. All the transfection experiments were performed with Lipofectamine 2000 Reagent. Luciferase levels were measured with a luciferase assay kit (Promega) in a luminometer. The signals were first calibrated according to the lysate protein concentration, and then normalized to the pGL3 transfected control group sample.

### Tumor Growth

All the animal-related experiments were performed following our protocol approved by the Institutional Animal Care and Use Committee of Zhoushan Hospital, Zhejiang University (Zhoushan, China). Briefly, A549 cells were injected subcutaneously into 6–8 week-old female athymic nude mice (Shanghai Laboratory Animal Center, Shanghai, China) to form xenograft tumors. One week after the injection was done, all the mice were randomly distributed into four groups (five mice per group). The mice were then injected with M2-derived exosomes once every 3 days and with either 5 mg/kg DDP (1 mg/ml, dissolved in PBS) or equal volume of PBS once a week as described previously (Lee and Schmitt, 2019). Tumor growth was monitored daily and tumors were measured every 3 days using digital calipers after the xenograft was established. Tumor size was defined by: volume = 0.5 × length × width^2^. The mice were euthanized 33 days after injection. All the tumors weight were measured by digital scales. Tumor tissue was cut into two halves, one half was fixed in 4% Formaldehyde solution to make paraffin embedded section for terminal deoxynucleotidyl transferase dUTP nick end labeling (TUNEL). The other half was snap frozen in liquid nitrogen for qPCR and western blotting analyses.

### Statistical Analysis

All the statistical analysis was performed using the Graphpad Prism software (version 6.0, San Diego, CA, USA). *P*-value of the experiments were analyzed by analysis of variance (ANOVA) or unpaired Student's *t*-test. *P* values < 0.05 were considered significant.

## Results

### M2 Macrophages Enhance Aerobic Glycolysis and Inhibit DDP-Induced Apoptosis in Lung Cancer

**Figure 1:**
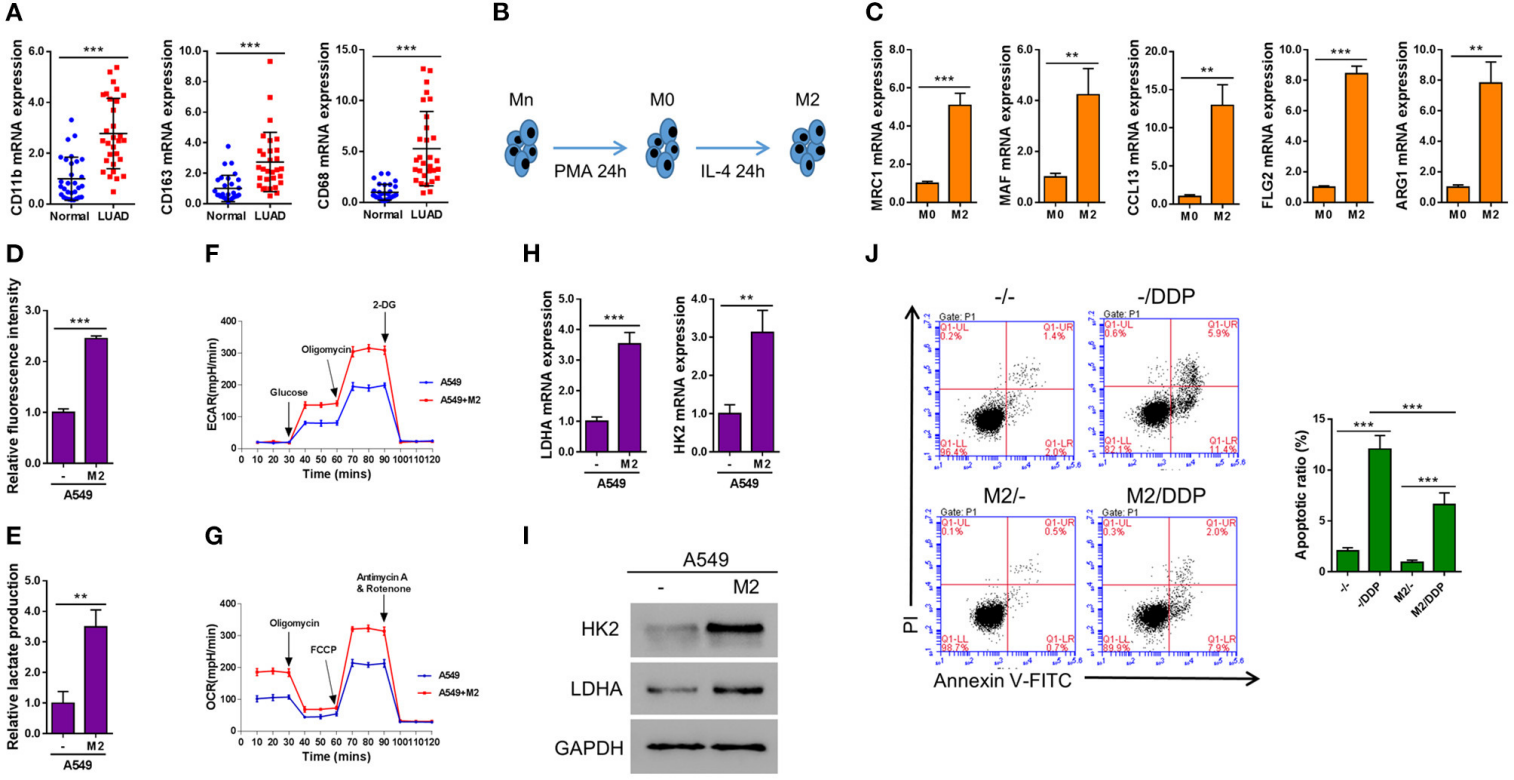
M2 macrophages enhance aerobic glycolysis and inhibit DDP-induced apoptosis in A549 cells. **(A)** Markers of M2 macrophages in LUAD tissues and adjacent normal tissue (*n* = 30). **(B)** A schematic diagram chart showing Mn THP-1 cells first were activated into M0 macrophages, and then polarized into M2 macrophages. **(C)** Real-time qPCR of the markers of polarization of M2 macrophages. **(D–J)** A549 lung cancer cells were seeded in the lower chamber, and M2 macrophages were seeded in transwell inserts. After 2 days, the inserts were removed. Induced glucose uptake **(D)**, lactate production **(E)**, ECAR **(F)**, and OCR **(G)** in A549 cells when cultured in conditioned media from M2 macrophages. Induction of HK2 and LDHA in A549 cells by co-culture with M2 macrophages in a Transwell system was analyzed by RT-qPCR **(H)** and western blotting **(I)**. Flow cytometry analysis for Annexin V-FITC and propidium iodide (PI) staining **(J)** in A549 cells co-cultured with M2 macrophages in a Transwell system and treated with or without Cisplatin (DDP, 10 μM) for 24 h. **P < 0.01, ***P < 0.001.

In order to determine the biological function of M2 macrophage in lung cancer tissue, we firstly assessed the status of M2 macrophage in LUAD samples. By determining the expression levels of three major M2 macrophage markers across LUAD samples as well as in adjacent normal control tissues, we found that compared with normal tissues, M2 macrophages were significantly enriched in LUAD tissues (Figure 1A). It is known that Mn THP-1 cells could be activated into M0 macrophages upon PMA stimulation for 24 h or be polarized into M2 macrophages after being treated with IL-4 for 24 h (Figure 1B). To characterize the polarized macrophages, the expression levels of multiple M2 macrophage markers including MRC1, MAF, CCL13, FLG2, and ARG1 were tested. As shown in Figure 1C, compared with M0 macrophages, all the markers have been upregulated, indicating that the Mn cells have been successfully polarized into M2 macrophages. In order to determine the effect of M2 macrophage on lung cancer cells in terms of metabolism, A549 lung cancer cells were co-cultured with M2 macrophages in a Transwell system for 2 days and then A549 cells were subjected to subsequently analyses. Interestingly, the glucose uptake (Figure 1D), lactate production (Figure 1E), as well as basal and maximal ECAR (Figure 1F) and OCR (Figure 1G) were significantly promoted when A549 cells were co-cultured with M2 macrophage. Consistent with this observation, the expression of two glycolysis-related genes, *LDHA* and *HK2*, were elevated at both mRNA as well as protein levels (Figures 1H,I). Next we aimed to determine whether M2 macrophages confer chemoresistance in lung cancer. As shown in Figure 1J, the proportion of apoptotic cells induced by DDP was significantly decreased in A549 cells co-cultured with M2 macrophages compared with A549 cells without being co-cultured with M2 macrophages. Taken together, our data demonstrated that M2 macrophage promoted aerobic glycolysis and the development of DDP-resistance in lung cancer cells.

### Exosomes Derived From M2 Macrophages Can Enhance Aerobic Glycolysis and Thereby Inhibit DDP-Induced Apoptosis in A549 Cells

**Figure 2:**
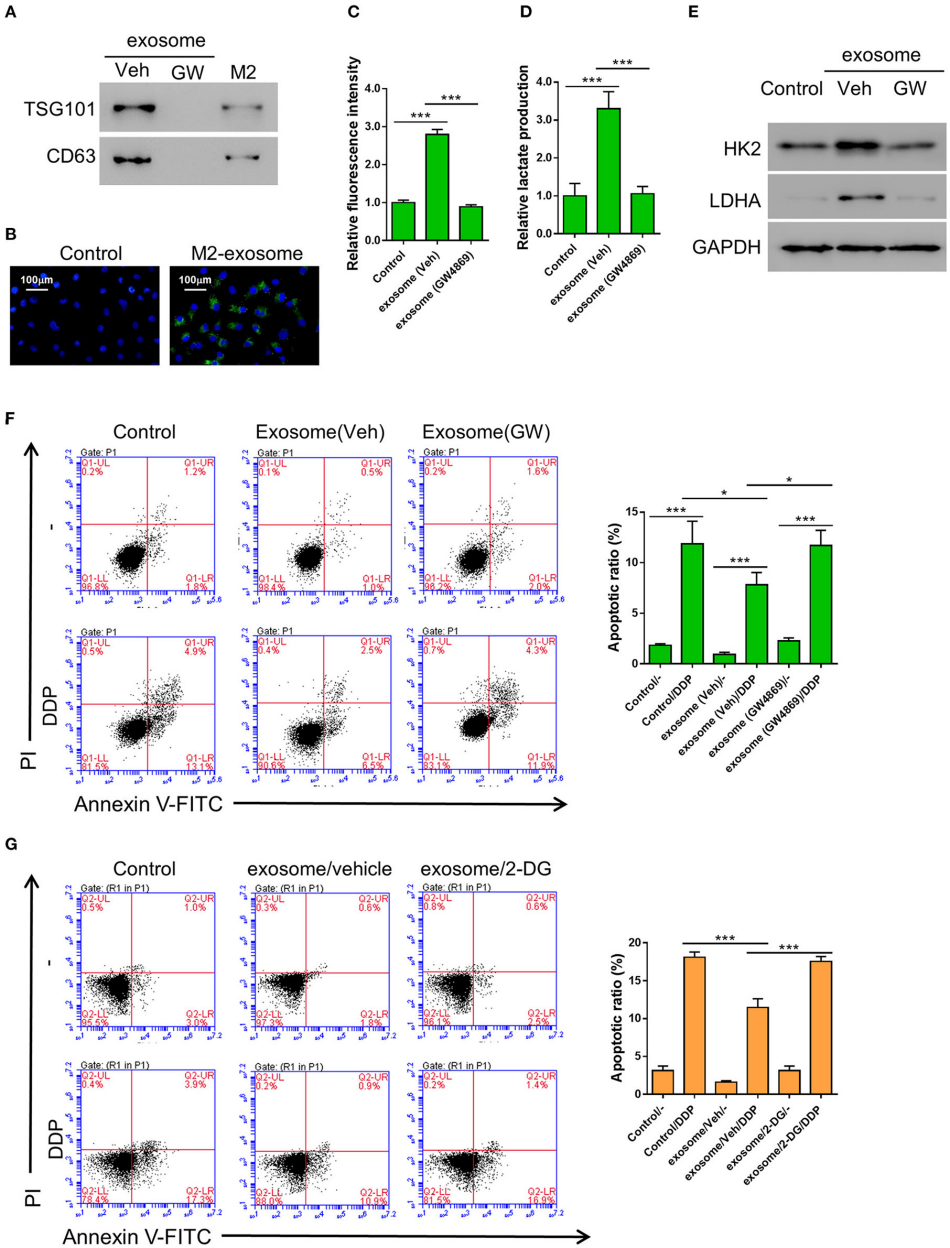
Exosomes from M2 macrophages enhance aerobic glycolysis and thereby inhibit DDP-induced apoptosis in A549 cells. **(A)** Expression of exosomal markers TSG101 and CD63 in M2 cellular protein and corresponding exosomes were analyzed by western blotting. Exosome inhibitor GW4869-treated exosomes were used as negative control. **(B)** The PKH-67 labeled exosomes can be internalized by A549 cells. **(C–F)** A549 cells were co-incubated with M2-derived exosomes that have been treated with either DMSO or GW4869, then both the uptake of glucose **(C)** and lactate production **(D)** were determined as described previously, and the expression level of HK2 and LDHA were tested by western blot **(E)**, The DDP-induced apoptosis in various condition as described were tested (**F)**. **(G)** A549 cells were incubated with 2-DG (20 mM) or Vehicle (DMSO), M2-derived exosomes, and treated or without Cisplatin (DDP, 10 μM) for 24 h. *P < 0.05, ***P < 0.001.

It has been reported by other groups that exosomes may regulate glycometabolism as well as chemoresistance through the transportation of functional small RNAs and/or proteins (Qin et al., 2017; Maia et al., 2018). In order to confirm whether the promotive effect of M2 macrophage on aerobic glycolysis and DDP-resistance in cancer cells was achieved through producing exosomes, we first tried to isolate exosome from the conditioned medium of M2 macrophages. We successfully purified exosomes, as evidenced by the expression of exosomal markers TSG101 and CD63, which could be abolished by the treatment of a widley-used exosome inhibitor GW4869 (Essandoh et al., 2015; Chen et al., 2019; Gu et al., 2019) (Figure 2A). The purified exosomes derived from M2 macrophage displayed as small round vesicles with the average diameter of either 58 ± 10 nm or 100 nm depending on the method utilized for particle size analysis (Supplementary Figure 1). These results demonstrate that M2 macrophages can produce exosomes. To examine if the exosomes can be taken up by lung cancer cells, PKH67-labeled exosomes were added to A549. As shown in Figure 2, the exosomes can be internalized by A549 cells after 24 h of incubation.

In order to confirm whether the M2 macrophage-derived exosomes are the main factors that promoted aerobic glycolysis in A549 cells, the cells were incubated with exosomes derived from either DMSO (the vehicle group) or GW4869-treated M2 macrophages, then the uptake of glucose and lactate production were evaluated as described above. As we expected, both the uptake of glucose and lactate production were significantly elevated by the exosomes derived from DMSO-treated M2 macrophages, but not by those derived from GW4869-treated M2 macrophages (Figures 2C,D). Similarly, the glycolysis-related gene, *HK2* and *LDHA*, were both upregulated by the exosomes derived from DMSO-treated M2 macrophages, but not by those derived from GW4869-treated M2 macrophages (Figure 2E). To further investigate the role of M2-derived exosomes in the DDP-resistance of lung cancer cells, A549 cells were first incubated with exosomes derived from either DMSO (the vehicle group) or GW4869-treated M2 macrophages, then treated with DDP. The flow cytometry assay showed that co-incubation with M2-derived exosomes significantly decreased DDP-induced apoptosis compared with controls (Figure 2F). To explore whether glycolysis affected chemoresistance, 2-DG, a glycolysis inhibitor (Abboud et al., 2018), was applied. A549 cells were first incubated with either DMSO (the vehicle group) or 2-DG, then treated with M2-derived exosomes and DDP. The flow cytometry assay showed that 2-DG restored DDP-induced apoptosis compared with cells treated with vehicle (Figure 2G).

Collectively, our data suggested that M2-derived exosomes are the main functional factor in M2 macrophages to promote aerobic glycolysis and chemoresistance in lung cancer cells.

### Functional miR-3679-5p Is Transmitted From Macrophages to Lung Cancer Cells Through M2-Derived Exosomes

**Figure 3:**
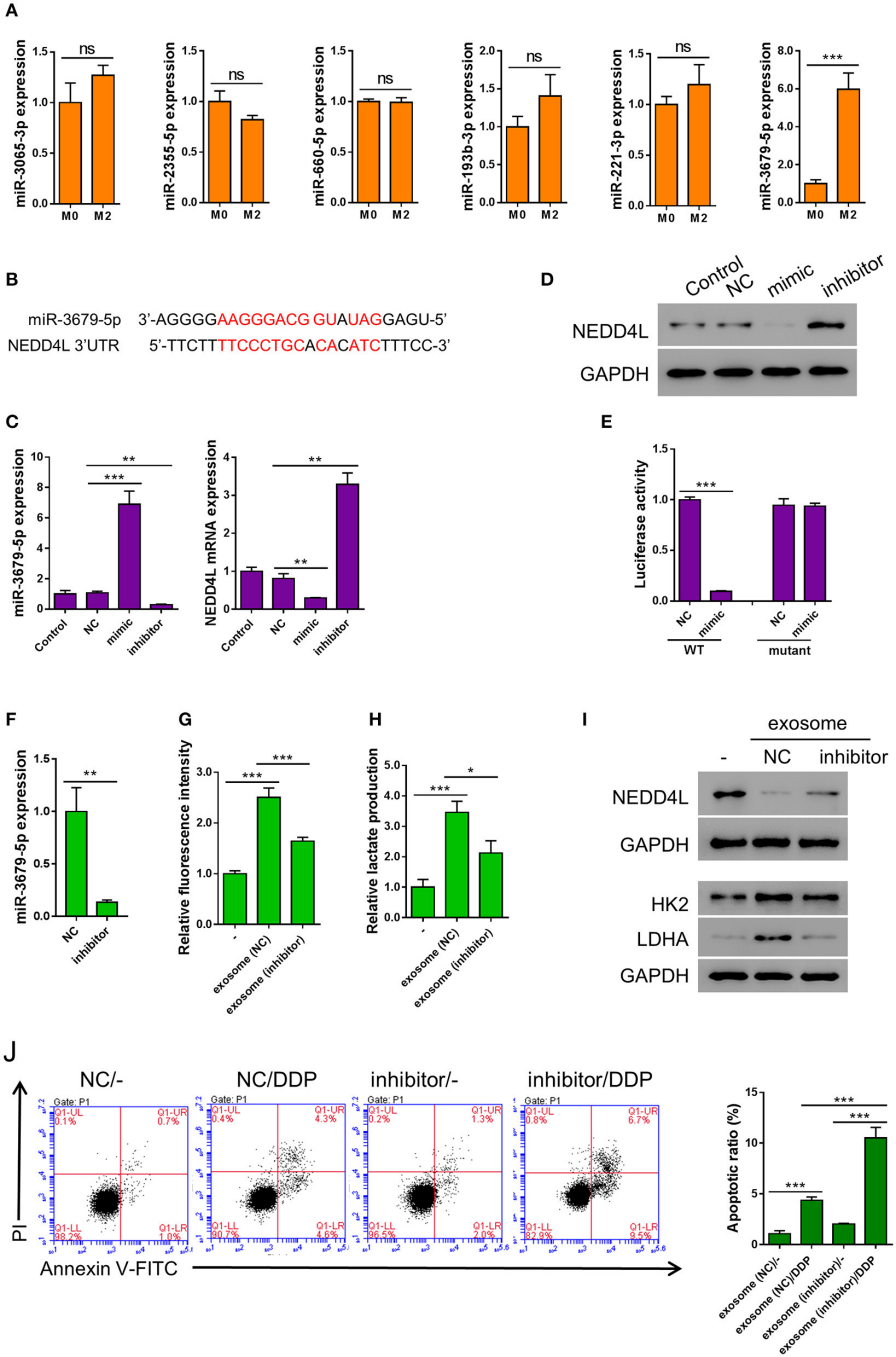
miR-3679-5p transmitted by M2 exosomes enhances aerobic glycolysis and induces apoptosis resistance in A549 cells. **(A)** Expression level of indicated miRNAs between M0-derived exosomes and M2-derived exosomes. **(B)** miR-3679-5p showed the most likely binding with the 3'UTR of NEDD4L as predicted by mirwalk database. **(C,D)** A549 cells were transfected with miR-3679-5p mimic, then treated with vehicle or miR-3679-5p-inhibitor. Then the mRNA **(C)** or protein **(D)** level were determined. **(E)** Luciferase reporter assay to show that miR-3679-5p could target NEDD4L on 3′UTR region. **(F)** Expression level of miR-3679-5p in exosomes derived from M2 transfected with either miR-3679-5p-inhibitor or NC control for 24 h. **(G–J)** A549 cell was co-cultured with M2-derived exosomes which has been transfected with either miR-3679-5p-inhibitor or NC control for 24 h, then the glucose uptake rate **(G)**, and lactate production **(H)** were determined. The protein level of NEDD4L, HK2, and LDHA were determined by western blot **(I)**, DDP-induced apoptosis was determined by flow cytometry **(J)**. Ns, not significant. *P < 0.05, **P < 0.01, ***P < 0.001.

Emerging evidence suggests that miRNAs are frequently encapsulated in exosomes and excute their biological functions in the donee cells to facilitate cell-cell communication (Maia et al., 2018). It has been reported that the expression of multiple miRNAs (miR-3065-3p, miR-2355-5p, miR-660-5p, miR-193b-3p, miR-221-3p, and miR-3679-5p) were elevated in M2 compared with M0 macrophages (Cobos Jimenez et al., 2014). To verify whether these miRNAs are also upregulated in the M2-derived exosomes, we generated the expression profiles of these miRNAs in the exosomes from both M0 and M2 macrophages. To our surprise, among the six miRNAs tested, only the expression of miR-3679-5p showed significant difference between M0 and M2 exosomes (Figure 3A). Prediction by mirwalk database showed that NEDD4L, a known E3 ubiquitin ligase, could be one target of miR-3679 (Figure 3B) (Sticht et al., 2018). Studies from other groups have shown that NEDD4L plays a critical role in tumorigenesis by regulating various downstream signaling pathways such as VEGFR2, MDM2, and pAKT (Murdaca et al., 2004; Fan et al., 2013; Xu et al., 2015). Furthermore, according to the RNAseq data from TCGA database, we found that NEDD4L expression was significantly reduced in LUAD samples compared with normal tissues (Supplementary Figure 2 left). In addition, lower expression level of NEDD4L was significantly correlated with worse survival in LUAD patients (Supplementary Figure 2 right).

In order to confirm whether NEDD4L is a target of miR-3679-5p in lung cancer, we transfected A549 cells with miR-3679-5p mimic, then the expression level of NEDD4L was determined by qRT-PCR and western blot. It could be seen in Figures 3C,D that the expression level of NEDD4L was downregulated by miR-3679-5p. This effect could be attenuated by the treatment of miR-3679-5p inhibitor (miRi). To further confirm whether the transcription of *NEDD4L* gene is directly regulated by miR-3679-5p, we co-transfected miR-3679-5p mimic and a luciferase reporter plasmid driven by either wild-type (WT) or mutant *NEDD4L*-3′UTR into A549 cells. The luciferase activity within the cells was then determined. According to our results, miR-3679-5p specifically inhibited the transcriptional activity of WT NEDD4L 3′UTR (Figure 3E). To further investigate whether miR-3679-5p is the only functional molecule in M2 exosomes that confers A549 cells enhanced aerobic glycolysis and DDP resistance, M2 exosomes were first transfected with miR-3679-5p inhibitor (Figure 3F), then co-cultured with A549 cells whose glucose uptake and lactate production were driven by MDE treatment. As we expected, the glycolysis and lactate production-inducing effects of MDE were both significantly reduced when the function of miR-3679-5p was inhibited (Figures 3G,H). Likewise, the expression of glycolysis-related genes as well as that of *NEDD4L* were all correlated with the existence of functional miR-3679-5p (Figure 3I). In addition, MDE-induced DDP-resistance was clearly abolished by the miR-3679-5p inhibitor (Figure 3J). In summary, these data suggest that miR-3679-5p is the main functional molecule in M2 macrophage-derived exosomes to regulate glycolysis and chemoresistance in lung cancer cells.

### M2 Macrophages Enhance Aerobic Glycolysis of Lung Cancer by Reducing NEDD4L-Mediated c-Myc Ubiquitination

**Figure 4:**
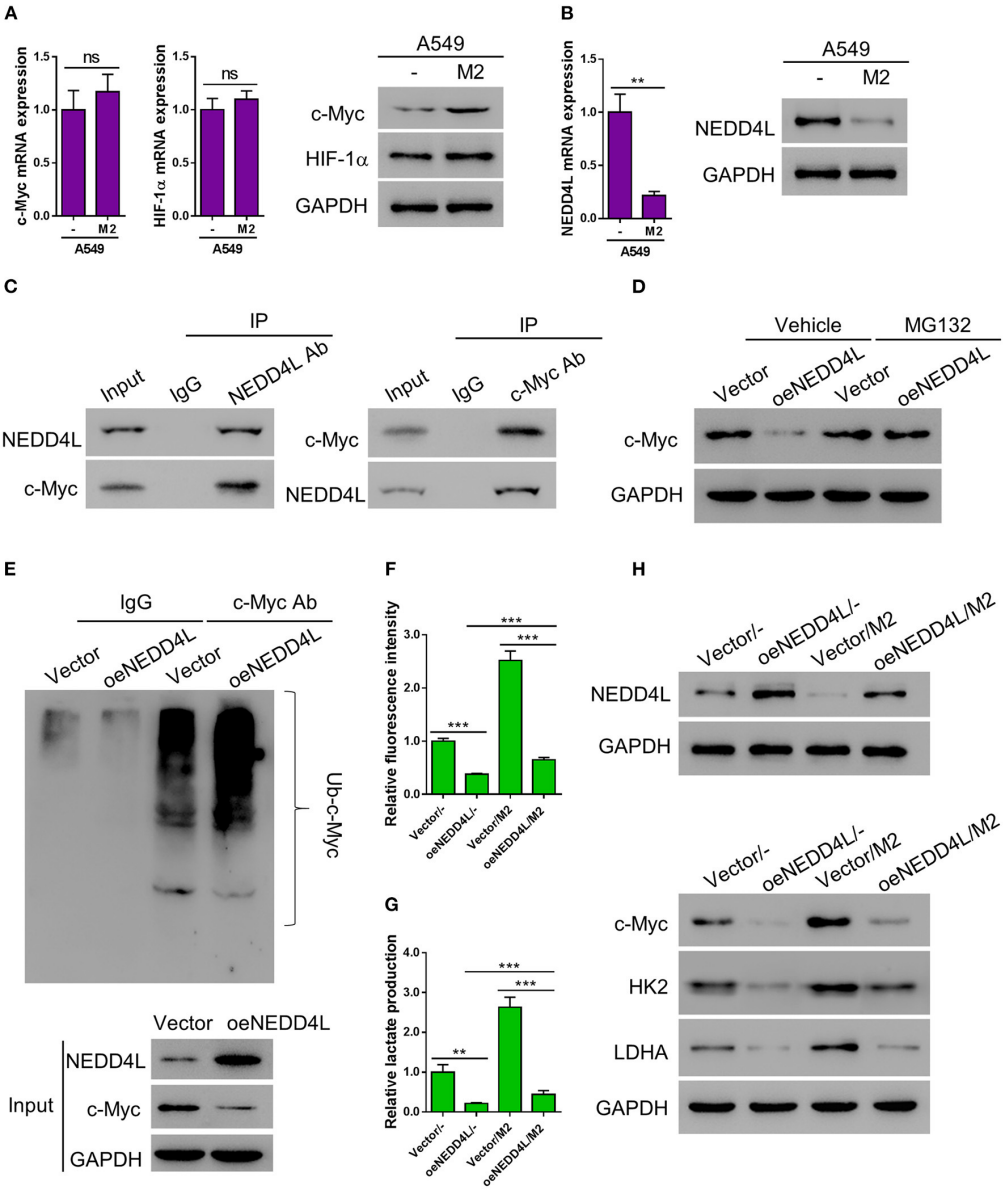
M2 macrophages enhance aerobic glycolysis of A549 cells by reducing NEDD4L-mediated c-Myc ubiquitination. **(A)** Expression of c-Myc and HIF-1α in A549 cells co-cultured with M2 macrophages in a Transwell system was analyzed by RT-qPCR (left panel) and western blotting (right panel). **(B)** Expression of NEDD4L in A549 cells co-cultured with M2 macrophages in a Transwell system was analyzed by RT-qPCR (left panel) and western blotting (right panel). **(C)** Co-IP assay to verify the interaction between NEDD4L and c-Myc in A549 cells. **(D)** A549 that overexpressing NEDD4L was treated with MG132 (10μM) for 24 h before the protein level of c-Myc was tested by western blot. **(E)** Cell lysate form HK2 overexpressing NEDD4L was subjected to immunoprecipitation assay against c-Myc, then the ubiquitination status of c-Myc was tested by western blot. **(F–H)** A549 cells overexpressing NEDD4L were co-cultured with M2 macrophages in Transwell system for 2 days. Then the analysis of glucose uptake **(F)** and lactate production **(G)** were performed. The expression of NEDD4L, c-Myc, HK2, and LDHA were determined by western blot **(H)**. **P < 0.01, ***P < 0.001.

According to the E3 ligase target prediction from the Ubibrowser online tool (Li et al., 2017), NEDD4L is an E3 ligase that can potentially target c-Myc (Supplementary Figure 3). Therefore, we performed RT-PCR and western blot to examine whether the expression of c-Myc was altered in A549 cells co-cultured with M2 macrophages. Interestingly, the protein level of c-Myc was elevated in A549 cells co-cultured with M2 macrophages, while the mRNA level of *c-Myc* gene was barely affected, suggesting that the expression of c-Myc in A549 cells is mainly regulated by M2 macrophage co-culture at the posttranslational level (Figure 4A). Meanwhile, consistent with our aforementioned observations, the expression of NEDD4L was significantly suppressed at both mRNA and protein levels in A549 cells co-cultured with M2 macrophage (Figure 4B). In order to verify whether NEDD4L is involved in regulating c-Myc protein stability, we firstly performed co-immunoprecipitation (Co-IP) assay to investigate the interaction between NEDD4L and c-Myc. As shown in Figure 4C, NEDD4L can physically interact with c-Myc in A549 cells. To further examine whether NEDD4L can regulate c-Myc protein stability, we established an A549 cell line that constitutively overexpresses NEDD4L (oeNEDD4L) (Supplementary Figure 4). We found that the protein level of c-Myc was dramatically reduced in the oeNEDD4L cell line. However, the protein level of c-Myc could be restored by the treatment of proteasome inhibitor MG132 (Figure 4D). Furthermore, we observed that c-Myc is evidently ubiquitinated in presence of the overexpressed NEDD4L (Figure 4E). These data suggest that c-Myc is a substrate of NEDD4L for ubiquitination in A549 cells. Our data showed that forced overexpression of c-Myc could dramatically increase the level of glucose uptake and lactate production in lung cancer cell (Supplementary Figure 5). In addition, NEDD4L overexpression could efficiently attenuate the M2-macrophage induced glycolysis in A549 cells (Figures 4F,G), and such effect could be achieved through the reduction of c-Myc protein level (Figure 4H). Taken together, reduction of NEDD4L-mediated c-Myc ubiquitination could be the mechanism by which M2 macrophages promote aerobic glycolysis in lung cancer cells.

### M2 Macrophage-Derived Exosomes Induce the Resistance of Lung Cancer to DDP *in vivo*

**Figure 5:**
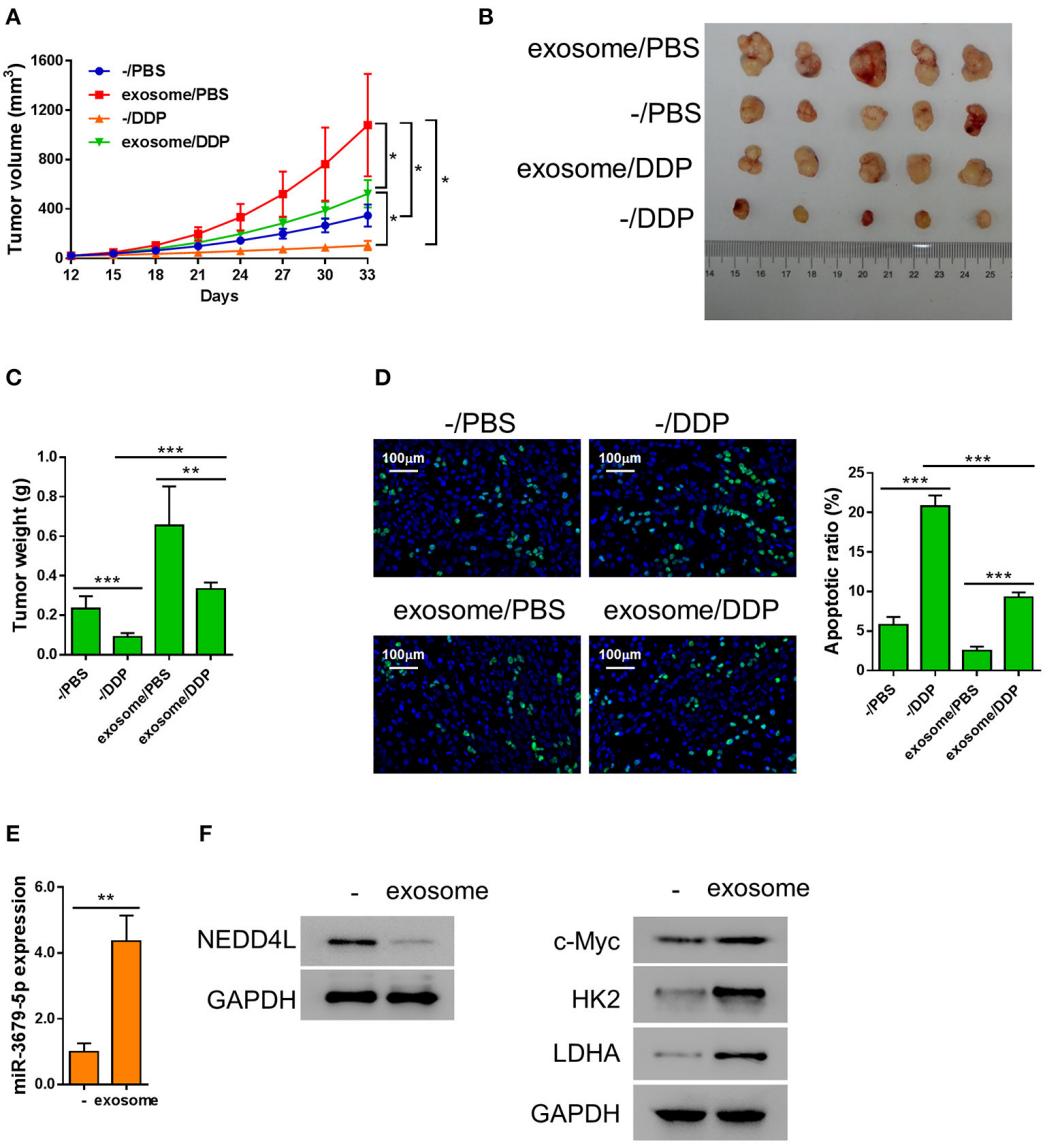
Exosomes from M2 macrophages induce chemoresistance in lung cancer *in vivo*. A549 cells were injected into nude mice subcutaneously, then the mice bearing xenografts were injected with MDE once every 3 days, and 5 mg/kg DDP weekly as described previously (Lee and Schmitt, 2019). **(A)** Growth curve of the A549 xenograft tumors receiving different treatments as describe in the chart. **(B,C)** Both the sizes and weights of representative tumors from each group were exhibited. **(D)** The status of apoptosis of tumors from each treatment group were determined by Tunel assay with fluorescence staining. **(E)** The expression of miR-3679-5pwas determined by qRT-PCR. **(F)** The expression of NEDD4, c-Myc, HK2, and LDHA was determined by western blot. *P < 0.05, **P < 0.01, ***P < 0.001.

We next investigated the function of M2 macrophage-derived exosomes in chemoresistance of lung cancer *in vivo*. Athymic nude mice bearing A549 xenograft tumors were injected with M2 exosomes every 3 days, followed by a weekly treatment of DDP intraperitoneally. We found that the tumor tissues from the exosome-treated group were significantly larger and heavier than those from the mock group under the circumstance of DDP treatment (Figures 5A–C), indicating that M2 macrophage-derived exosomes significantly suppressed the chemotherapeutic effect of DDP. We then evaluated the apoptosis status of the xenografts by TUNEL assay and found that M2 macrophage-derived exosomes dramatically reduced DDP-induced apoptosis *in vivo* (Figure 5D). In addition, among the members of the miR-3679-5p/NEDD4L/c-Myc regulatory axis, miR-3679-5p was significantly enriched in exosome-treated xenograft tumors (Figure 5E), the protein level of NEDD4L was evidently downregulated, while the protein level of c-Myc was significantly increased (Figure 5F). As a result, the expression levels of glycolysis-related genes were upregulated (Figure 5F). Taken together, we demonstrate that M2 macrophage-derived exosomes promoted glycolysis and conferred lung tumor DDP resistance through the miR-3679-5p/NEDD4L/c-Myc regulatory axis.

### The Expression of NEDD4L Is Negatively Correlated With Those of c-Myc and miR-3679-5p in LUAD Samples

**Figure 6:**
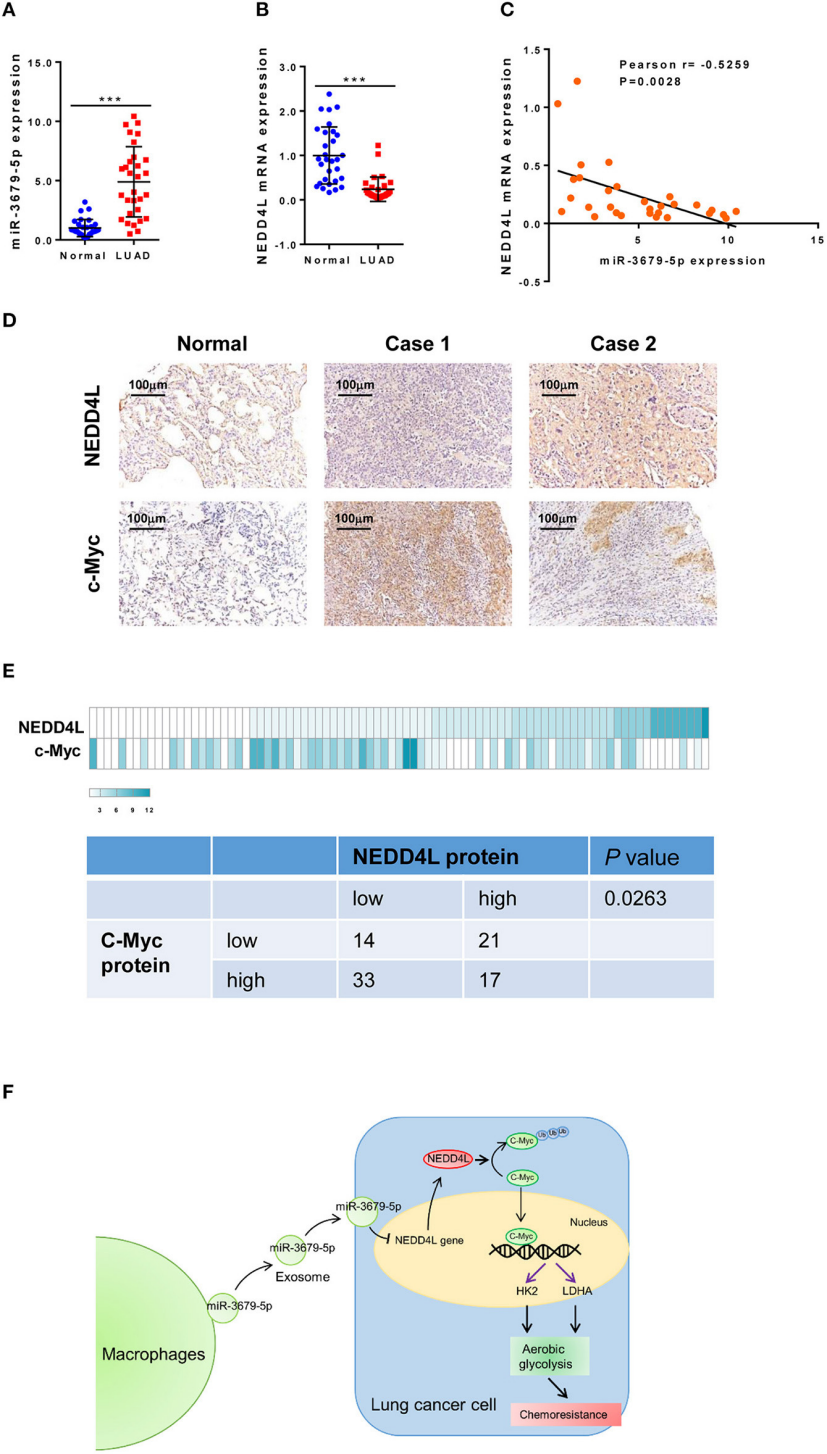
Correlation analysis of miR-3679-5p and NEDD4L in LUAD tissues. **(A,B)** Expression of miR-3679-5p and NEDD4L in LUAD tissues and adjacent normal tissue were shown in dot plots (*n* = 30). **(C)** Pearson correlation between NEDD4L expression and miR-3679-5p in LUAD tissues was shown in scatter plots (*n* = 30). **(D,E)** NEDD4L and c-Myc expression in LUAD tissues by IHC staining. Representative images of IHC staining section **(D)** were shown. Heatmap of The heatmap represented the protein expression quantity of NEDD4L and c-Myc (upper panel). Fisher's exact test was shown in the lower panel. **(F)** Schematic illustration of MED mediating miR-3679-5p/NEDD4L/c-Myc signal axis and driving enhanced aerobic glycolysis as well as chemoresistance in lung cancer cells.

In order to confirm our *in vitro* and *in vivo* findings, we analyzed the expression pattern of miR-3679-5p and NEDD4L in LUAD samples. Compared with normal tissues, the expression of miR-3679-5p was significantly increased, while the expression of NEDD4L was significantly decreased in LUAD samples (Figures 6A,B). The correlation between the expression of miR-3679-5p and NEDD4L was analyzed using the Pearson r correlation test based on the TCGA database. We observed a significant negative correlation between the expression of miR-3679-5p and that of NEDD4L (Figure 6C). Next, we measured the protein levels of NEDD4L and c-Myc in LUAD tissues by IHC staining. The staining intensity (SI) of the protein was stratified as weak, moderate, and strong. Our data showed that protein level of c-Myc was negatively correlated with that of NEDD4L in LUAD samples (Figures 6D,E). Together, these results confirmed our *in vitro* and *in vi*vo findings by showing that the expression of NEDD4L is negatively correlated with those of c-Myc and miR-3679-5p in LUAD samples.

## Discussion

Cisplatin is considered as one of the first-line chemotherapy reagents in lung cancer treatment. Unfortunately, advanced lung cancer patients tend to develop resistance to cisplatin, which leads to poor prognostic outcome. In addition to genetic changes that directly confer drug resistance in cancer cells, tumor microenvironment factors could also protect cancer cells from being killed by chemotherapy (Klemm and Joyce, 2015). Therefore, it is essential to study the mechanisms causing drug resistance to improve lung cancer treatment. Accumulated evidence suggests that exosomes in cancer function as mediators that transfer functional biological materials, including proteins, DNA, and RNA, via cell-cell communication (Samir et al., 2013). It has been reported that exosomes play critical roles in the development of chemoresistance in cancer (Boelens et al., 2014; Sousa et al., 2015; Qu et al., 2016). In the present study, we demonstrated that the exosomes derived from M2 macrophages induce cisplatin-resistance in lung cancer both *in vitro* and *in vivo* via a miR-3679-5p/NEDD4L/c-Myc regulatory axis.

The role of miRNA in the development of cancer has gained increasing attention. A great number of studies have described a variety of roles of miRNAs in chemoresistance (Wang et al., 2011, 2014; Zhang et al., 2012; Lei et al., 2013; Li et al., 2013, 2014; Dong et al., 2014; Ning et al., 2014; Yu et al., 2014; Ma et al., 2015; Zhao et al., 2015). M2a-polarized macrophages have changed miRNA expression signature during their maturation. It has been reported that multiple known and potential tumor suppressors including miR-3065-3p, miR-2355-5p, miR-3679-5p, miR-660-5p, miR-193b-3p, and miR-221-3p are upregulated in M2a compared with human monocytes (Cobos Jimenez et al., 2014). All these miRNAs, without exception, could potentially suppress the expression of NEDD4L according to the miRNA target prediction algorithm. NEDD4L is a member of the NEDD4 family of HECT domain E3 ubiquitin ligases, which label target proteins for lysosomal degradation (Ingham et al., 2004). The protein is ubiquitously expressed in human body. It has been demonstrated that the NEDD4 family plays a vital role in the development and progression of human cancers, and that the expression of NEDD4L is downregulated in various cancer types. For instance, the study from Hu et al. suggested that NEDD4L expression is suppressed in prostate cancer compared with benign prostatic hyperplasia (Hu et al., 2009). Tanksley et al. have found that the expression of NEDD4L is downregulated in colorectal cancer, which results in inhibited canonical WNT signaling pathway (Tanksley et al., 2013). Our data suggest that NEDD4L is negatively regulated by miR-3679-5p shuttled by M2 macrophage-derived exosomes. As an E3 ligase, NEDD4L exerts its biological functions mainly through mediating target protein ubiquitination and degradation in endoplasmic reticulum or lysosomes, or by proteasome (Shearwin-Whyatt et al., 2006). In the current study, we demonstrated that NEDD4L regulates the stability of c-Myc through mediating its ubiquitination. As a well-studied proto-oncogene, c-Myc has been demonstrated to play multiple roles in tumor development. Furthermore, c-Myc has been reported to regulate cellular sensitivity to DDP (Torigoe et al., 2005; Xie et al., 2014; Reyes-Gonzalez et al., 2015). We speculate that c-Myc could contribute to the acquired DDP-resistance in A549 lung cancer cells. This hypothesis has been backed up by other studies, for instance, Zuo et al. have reported that c-Myc is significantly overexpressed in A549/DDP cell line, which is a DDP-resistant subclone of A549 cell line. On the other hand, it has been reported that exosome exchange between tumor cells and macrophages is bidirectional (Maia et al., 2018). In this study, we did not examine exosomes derived from the lung cancer cells to macrophage. We have showed that macrophage could promote DDP-resistance in lung cancer, but not the other way around, which could be equally important to fully understand the crosstalk between tumor and microenvironment. We could in the future focus on the study to investigate how lung cancer cells educate macrophage into a supportive partner. In addition, some limitations existed in this study. First, only one NSCLC cell and one macrophage cell line were used. Second, the expression of miR-3679-5p in lung cancer specimens was not detected. Further exploration is required to strength our current findings.

In conclusion, our results shed light on the mechanism of how exosomes contribute to the development of DDP resistance in lung cancer. As shown in Figure 6F, miR-3679-5p was delivered from M2a macrophages to lung cancer cells through internalization of exosomes. miR-3679-5p then inhibits the transcription of NEDD4L, a E3-ligase that regulates the ubiquitination and degradation of c-Myc. Stabilized c-Myc promoted aerobic glycolysis and induced chemoresistance in lung cancer cells. By identifying the miR-3679-5p/NEDD4L/c-Myc regulatory axis, we provided an insight into how DDP-resistance is developed in lung cancer cells. Therefore, our study provides the scientific basis for development of novel treatments for lung cancer.

## Data Availability Statement

The original contributions presented in the study are included in the article/Supplementary Material, further inquiries can be directed to the corresponding author/s.

## Ethics Statement

The studies involving human participants were reviewed and approved by the Institutional Ethical Review Committee of Zhoushan Hospital, Zhejiang University (Zhoushan, China). The patients/participants provided their written informed consent to participate in this study. The animal study was reviewed and approved by the Institutional Animal Care and Use Committee of Zhoushan Hospital, Zhejiang University (Zhoushan, China).

## Author Contributions

HW, XP, and ZC designed the experiments. HW, LW, HP, YW, and MS performed the experiments. HW, LW, HY, and CW performed the statistical analysis. HW and LW wrote the manuscript. XP and ZC supervised the study. All authors have read and approved the final version of the manuscript.

## Conflict of Interest

The authors declare that the research was conducted in the absence of any commercial or financial relationships that could be construed as a potential conflict of interest.
