# Supplementary material for: Exosomes Derived From Macrophages Enhance Aerobic Glycolysis and Chemoresistance in Lung Cancer by Stabilizing c-Myc via the Inhibition of NEDD4L
Source: Front Cell Dev Biol. 2021 Mar 4;8:620603. doi: 10.3389/fcell.2020.620603 (PMC7969980; doi:10.3389/fcell.2020.620603)
Supplement: Supplementary file 1 [file Data_Sheet_1.DOCX]

**
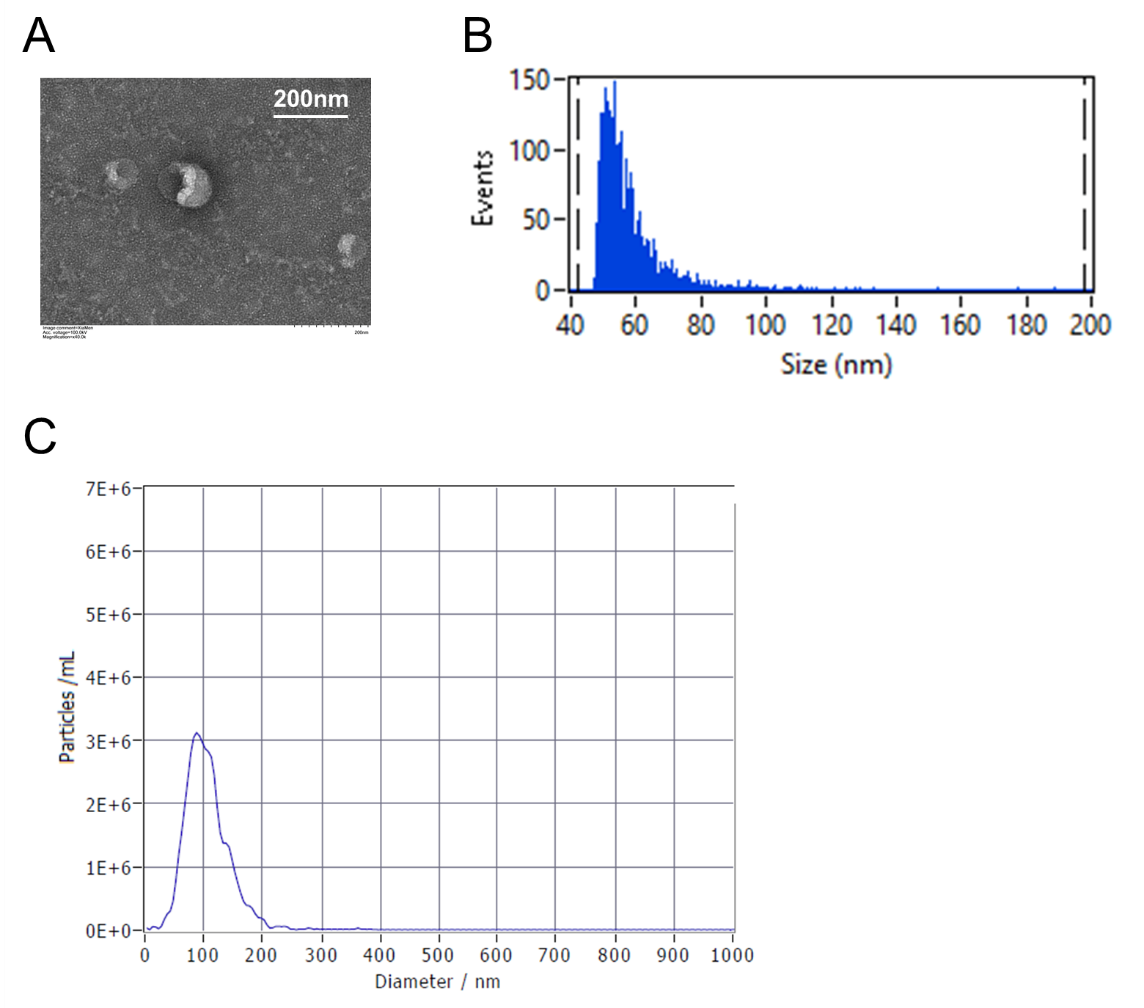
**

**Figure S1.** Characterization of exosomes from M2 macrophages. (A) Electron micrograph image. (B) Nanoanalyzer analysis of particle size distribution of exosomes, and the average diameter of exosomes was 58 ± 10 nm. (C) Particle size distribution in purified pellets consistent with size range of exosomes (average size 100 nm), measured by ZetaView® Particle Tracking Analyzer.


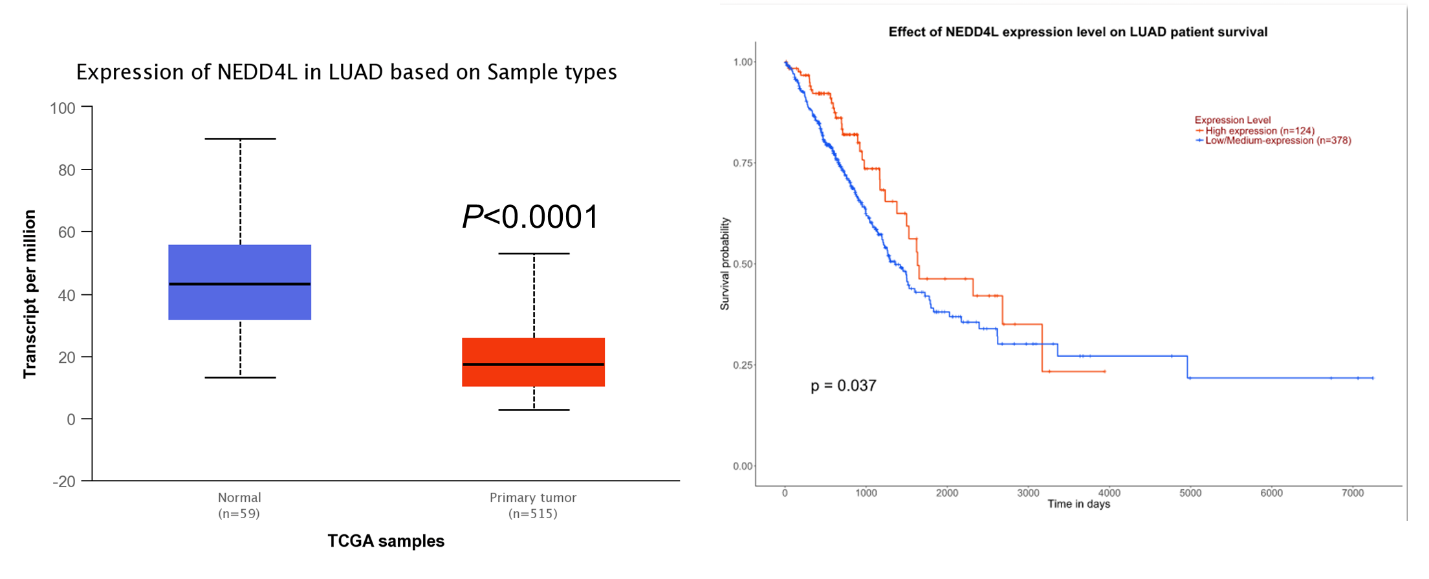


**Figure S2.** Gene expression and prognostic value of NEDD4L mRNA expression were performed based on The Cancer Genome Atlas (TCGA) lung adenocarcinoma (LUAD) dataset (http://ualcan.path.uab.edu/analysis.html). (A) The expression of NEDD4L is significantly lower in LUAD tumor tissue comparing to normal tissue in TCGA LUAD samples. (B) Kaplan-Meier plots overall survival with log-rank tests between NEDD4L high and low samples. The prognostic outcome of NEDD4L-low group was significantly worse compared with that of NEDD4L-high group.

**
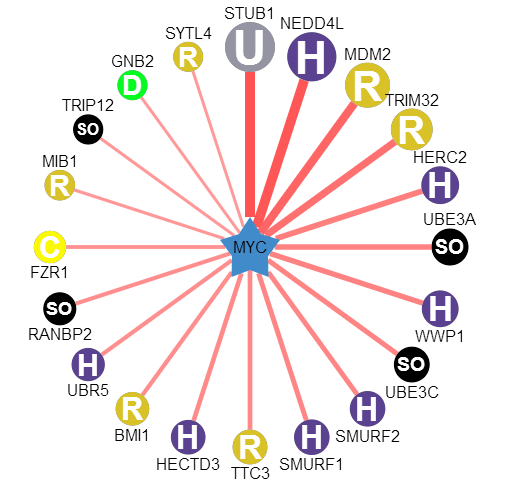
**

**Figure S3.** Prediction of candidate E3 ligase that could target c-Myc through a website <http://ubibrowser.ncpsb.org/ubibrowser/>.


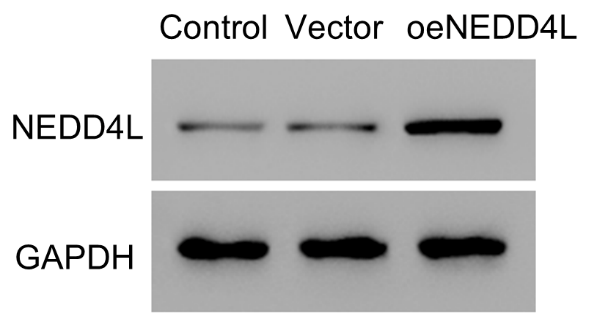


**Figure S4.** Overexpression of NEDD4L in A549 cells. The A549 infected with either oeNEDD4L or Vector control were subjected to western blot to validate the overexpression efficacy of NEDD4L.


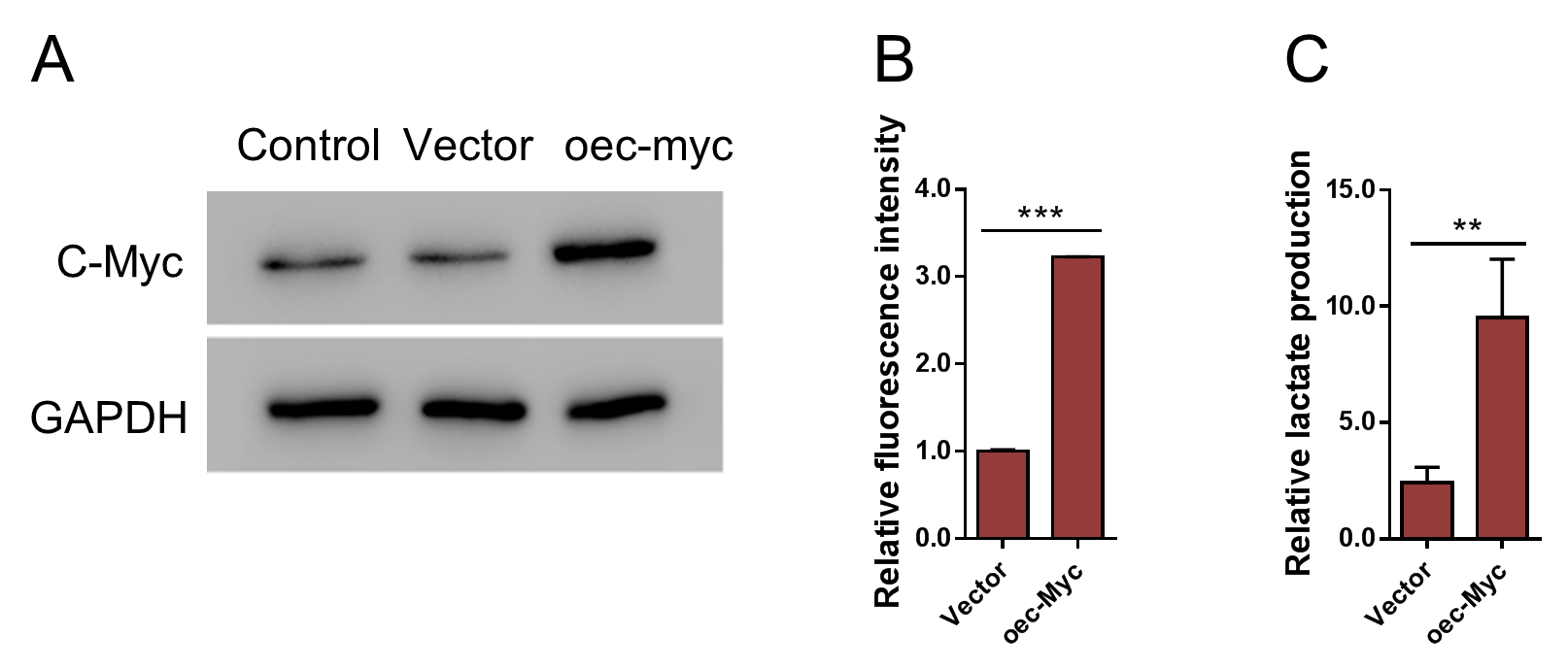


**Figure S5.** Forced overexpression of c-Mycpromoted aerobic glycolysis in A549. (A) The A549 cells infected with either oec-Myc or Vector control were subjected to western blot to validate the overexpression efficacy of c-Myc. Induced glucose uptake (B) and lactate production (C) in A549 cells overexpressing c-Myc.
