# Supplementary material for: Exosomes Derived From Macrophages Enhance Aerobic Glycolysis and Chemoresistance in Lung Cancer by Stabilizing c-Myc via the Inhibition of NEDD4L
Source: Front Cell Dev Biol. 2021 Mar 4;8:620603. doi: 10.3389/fcell.2020.620603 (PMC7969980; doi:10.3389/fcell.2020.620603)
Supplement: Supplementary file 2 [file Data_Sheet_2.DOCX]

**Table S1.** Primer sequences for real-time PCR.

| **Gene** | **Forward primer** | **Reverse primer** |
| --- | --- | --- |
| CD11b | 5' AGGGAGTCATTCGCTACG 3' | 5' TCAAAGTTATTCACCTGGAAC 3' |
| CD68 | 5' TGGCGGTGGAGTACAATG 3' | 5' GATCAGGCCGATGATGAG 3' |
| CD163 | 5' ATGTGGAGTTGCCCTTTC 3' | 5' GGACATAATGAAGCACCTAG 3' |
| MRC1 | 5' TGGGTGTCCGAATCTCAG 3' | 5' ACCCGATCCCTTGTAGAG 3' |
| MAF | 5' GAACTGGCAATGAGCAACTCCG 3' | 5' TTCCAGGTGCGCCTTCTGC 3' |
| CCL3 | 5' CTTCTGTGCCTGCTGCTC3' | 5' AATTCTGGACCCACTTCTC 3' |
| FLG2 | 5' CAAGTTGGAGTGAGGGAG 3' | 5' GAGCTGGAACCATGTCTG 3' |
| ARG1 | 5' GACCTGCCCTTTGCTGAC 3' | 5' TTCCGTTCTTCTTGACTTCTGC3' |
| NEDD4L | 5’ GACATGGAGCATGGATGGGAA3’ | 5’GTTCGGCCTAAATTGTCCACT3’ |
| GAPDH | 5' GGATTGTCTGGCAGTAGCC 3' | 5' ATTGTGAAAGGCAGGGAG 3' |
| HK2 | 5' GGCTCCAACGAGTTACCG 3' | 5' CACTTTGCCCATTTCAGG 3' |
| LDHA | 5' TCAGCAAGAGGGAGAAAG 3' | 5' CAAGCCACGTAGGTCAAG 3' |

**Table S2** Primer sequences for miRNA.

| **miRNA** | **Primers** |
| --- | --- |
| miR-3065-3p | RT-Primer：5' GTCGTATCCAGTGCAGGGTCCGAGGTATTCGCACTG GATACGACCTCCAA 3'  PCR Primer： 5' GCGTCAGCACCAGGATATTG 3' and 5' AGTGCAGGGTCCGAGGTATT 3' |
| miR-2355-5p | RT-Primer：5' GTCGTATCCAGTGCAGGGTCCGAGGTATTCGCACTGG ATACGACTTGTCC 3'  PCR Primer： 5' CGCGATCCCCAGATACAAT 3' and 5' AGTGCAGGGTCCGAGGTATT 3' |
| miR-3679-5p | RT-Primer：5' GTCGTATCCAGTGCAGGGTCCGAGGTATTCGCACTGGA TACGACTCCCCT 3'  PCR Primer： 5' CGTGAGGATATGGCAGGGA 3' and 5' AGTGCAGGGTCCGAGGTATT 3' |
| miR-660-5p | RT-Primer：5' GTCGTATCCAGTGCAGGGTCCGAGGTATTCGCACTGGA TACGACCAACTC3'  PCR Primer： 5' CGCGTACCCATTGCATATCG 3' and 5' AGTGCAGGGTCCGAGGTATT 3' |
| miR-193b-3p | RT-Primer：5' GTCGTATCCAGTGCAGGGTCCGAGGTATTCGCACTGG ATACGACAGCGGG 3'  PCR Primer： 5' GCGAACTGGCCCTCAAAGT 3' and 5' AGTGCAGGGTCCGAGGTATT 3' |
| miR-221-3p | RT-Primer：5' GTCGTATCCAGTGCAGGGTCCGAGGTATTCGCACTGG ATACGACGAAACC3'  PCR Primer： 5' CGCGAGCTACATTGTCTGCTG 3' and 5' AGTGCAGGGTCCGAGGTATT 3' |
| U6 | 5' CTCGCTTCGGCAGCACA 3' and 5' AACGCTTCACGAATTTGCGT 3' |

**Table S3.** Antibody list.

| **Primary antibody** | **Company** | **Catalog No.** |
| --- | --- | --- |
| HK2 | Abcam | Ab104836 |
| LDHA | Abcam | Ab101562 |
| c-myc | Abcam | Ab39688 |
| HIF-1α | Abcam | Ab16066 |
| NEDD4L | Abcam | Ab46521 |
| TSG101 | Abcam | Ab125011 |
| CD63 | Abcam | Ab118307 |
| GAPDH | Cell Signaling Technology | #5174 |
| Ubiquitin | Abcam | Ab7780 |
